# Genomic copy number variation analysis in multiple system atrophy

**DOI:** 10.1186/s13041-017-0335-6

**Published:** 2017-11-29

**Authors:** Yuka Hama, Masataka Katsu, Ichigaku Takigawa, Ichiro Yabe, Masaaki Matsushima, Ikuko Takahashi, Takayuki Katayama, Jun Utsumi, Hidenao Sasaki

**Affiliations:** 1 Department of Neurology, Faculty of Medicine and Graduate School of Medicine, Hokkaido University, Kita-15 Nishi-7, Kita-ku, Sapporo, 060-8638 Japan; 2 Mitsubishi Tanabe Pharma Corporation, 1000, Kamoshida-cho, Aoba-ku, Yokohama, 227-0033 Japan; 3 Graduate School of Information Science and Technology, Hokkaido University, Kita-14 Nisi-9, Kira-ku, Sapporo, 060-0814 Japan; 4 Division of Neurology, First Department of Internal Medicine, Asahikawa Medical University, 1-1, Higashi 2-jo 1-chome, Midorigaoka, Asahikawa, 078-8510 Japan

**Keywords:** Genomic DNA, Copy number variation, Multiple system atrophy, Array-comparative genome hybridization

## Abstract

Genomic variation includes single-nucleotide variants, small insertions or deletions (indels), and copy number variants (CNVs). CNVs affect gene expression by altering the genome structure and transposable elements within a region. CNVs are greater than 1 kb in size; hence, CNVs can produce more variation than can individual single-nucleotide variations that are detected by next-generation sequencing. Multiple system atrophy (MSA) is an α-synucleinopathy adult-onset disorder. Pathologically, it is characterized by insoluble aggregation of filamentous α-synuclein in brain oligodendrocytes. Generally, MSA is sporadic, although there are rare cases of familial MSA. In addition, the frequencies of the clinical phenotypes differ considerably among countries. Reports indicate that genetic factors play roles in the mechanisms involved in the pathology and onset of MSA. To evaluate the genetic background of this disorder, we attempted to determine whether there are differences in CNVs between patients with MSA and normal control subjects. We found that the number of CNVs on chromosomes 5, 22, and 4 was increased in MSA; 3 CNVs in non-coding regions were considered risk factors for MSA. Our results show that CNVs in non-coding regions influence the expression of genes through transcription-related mechanisms and potentially increase subsequent structural alterations of chromosomes. Therefore, these CNVs likely play roles in the molecular mechanisms underlying MSA.

**Electronic supplementary material:**

The online version of this article (10.1186/s13041-017-0335-6) contains supplementary material, which is available to authorized users.

## Introduction

Multiple system atrophy (MSA) is an adult-onset neurodegenerative disorder that is sporadic and progressive in nature. The estimated incidence is approximately 0.6 per 100,000 individuals per year; this increases to 3 per 100,000 per year in individuals older than 50 years, with a mean age of onset of approximately 60 years [1, 2]. The main clinical features comprise autonomic failure, cerebellar ataxia, levodopa-resistant Parkinsonism, and pyramidal signs with various combinations. According to the predominant motor presentations at examination, the phenotype of MSA is classified as Parkinsonian type (MSA-P) or cerebellar type (MSA-C) [3]. Pathologically, the brains of individuals with MSA exhibit atrophy of the cerebellum, brainstem, and posterolateral putamen, as well as the loss of pigmented neurons in the substantia nigra [1]. The neuropathological hallmark of MSA is the appearance of glial cytoplasmic inclusions, which are mainly composed of insoluble filamentous α-synuclein in oligodendrocytes. Lewy bodies, which also consist of α-synuclein, are found in Parkinson’s disease and in dementia with Lewy bodies, but not in MSA. These three disorders, including MSA, are classified as “synucleinopathies” [4, 5]. α-Synuclein is expressed predominantly in neurons, but it is not normally expressed in mature human oligodendrocytes [6–8]. Recently, it was reported that α-synuclein derived from neurons is released into the extracellular space from exosomes, or through exocytosis, and is incorporated into oligodendroglia [9]. Furthermore, in vitro studies showed that mutant fibrils of α-synuclein promote the fibrilization of α-synuclein and undergo cell-to-cell propagation [10, 11]. Growing evidence suggests that α-synuclein acts in a prion-like manner. However, the origin of α-synuclein in oligodendrocytes and its role in the misfolding or aggregation process in MSA have not been clearly defined.

As described in the 2nd Consensus Criteria, MSA constitutes a sporadic, non-hereditary disorder, although rare cases with familial history of MSA have been reported [3, 12, 13]. The frequencies of phenotypes in MSA vary among different ethnic groups. MSA-P is more common than MSA-C in Europe (62% vs 38%) and North America (60% vs 13%, the remaining 27% presenting with a mixed type) [14, 15]. In contrast, MSA-C is more common than MSA-P in Japan (67% vs 33%) [16, 17]. Thus, genetic predisposition to MSA has been suggested and evaluated. Genetic variations in the α-synuclein, beta-glucosylceramidase, or coenzyme Q2 genes, and individual single-nucleotide polymorphisms (SNPs) have been reported as risk factors for MSA; however, their roles in the etiology of MSA are not fully understood [18–21].

Genomic rearrangements in humans include duplications, deletions, insertions, inversions, and translocations. Structural variations, known as copy-number variations (CNVs), define genomic rearrangements over a range of 1 kb to several Mb. As CNVs cover such a large region of the genome, they may include entire genes and their regulatory regions [22]. In addition, CNVs occur in the presence of specific genomic structures that are highly susceptible to rearrangements, such as region-specific repeat sequences or low copy repeats [23]. Variable structural changes or rearrangements by CNVs directly or indirectly alter gene expression [23, 24].

Genetic disorders lacking heritability may result from low-frequency variants or de novo mutations with low or intermediate penetrance [24]. Through structural changes in genomes, CNVs affect gene dosage, gene expression, and expressed protein structures. Furthermore, the mutation rates of CNVs are at least 100–10,000-fold greater than those of point mutations [25]. Various studies have shown that CNVs are associated with human disease and play an important role in neurodevelopmental, neurodegenerative, and mental health disorders [23, 26, 27]. High-resolution genome-wide association studies, which are powerful tools for detecting disease-related genes, have enabled the identification of numerous single-gene Mendelian disorders. Various SNPs related to MSA have been identified via genome-wide association studies, although these only partially explain the etiology of the disorder [21]. In a study of a monozygotic twins discordant for MSA, i.e., in which one subject was affected by MSA and the other was not, we observed that copy number loss of the *SHC2* gene was related to MSA [28]. However, subsequent analysis did not confirm this result [29]. In the present study, we focused on CNVs rather than on SNPs to verify the relationship between MSA and CNVs, and attempted to evaluate the genetic heterogeneity in MSA using array-based comparative genomic hybridization (array-CGH).

## Results

### Identification of CNVs in 72 individuals

**Fig. 1:**
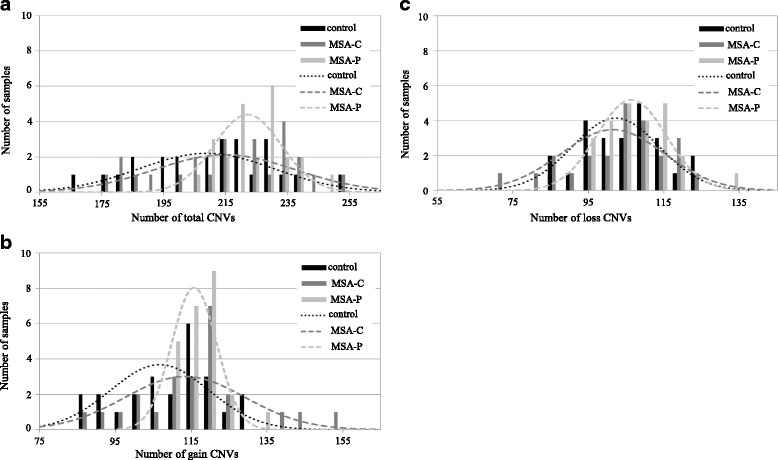
Histogram and distribution curve of each individual CNV in subjects with MSA-C or MSA-P and in controls. **a** Number of total CNVs (**b**) Number of gain CNVs (**c**) Number of loss CNVs

We assayed genomic DNA from 48 Japanese patients with MSA and 24 healthy Japanese controls for genome-wide CNV screening using Agilent 2x400K CNV arrays; the details of the subjects are described as the first set in Table 1. Microarray data were filtered with standard aberration filters and processed. A total of 15,499 autosomal CNVs were detected, 8059 (52%) of which comprised gain CNVs and 7440 (48%) of which were loss CNVs. We compared the numbers of total, gain, and loss CNVs in individuals with MSA-C and MSA-P and in the controls (Fig. 1a–c). The average numbers of CNVs in individuals were 209 in control subjects (*n* = 24), 215 in those with MSA-C (n = 24), and 222 in those with MSA-P (n = 24). The number of total and gain CNVs was significantly higher in individuals with MSA-P (*p* = 0.02, *p* = 0.01; Wilcoxon rank-sum test) compared to that in the control group. In contrast, the number of loss CNVs did not differ between groups. The variance of CNVs in MSA-C was nearly the same as that in the control group, although the number was slightly higher (Additional file 1: Table S1).

**Table 1:** Age and gender of Japanese participants

		1st set			2nd set		Validation set	
		MSA-C	MSA-P	Control	MSA-C	Control	MSA	Control
Number of subjects		24	24	24	40	40	245	212
Gender (M/F)		12/12	12/12	12/12	20/20	20/20	110/135	120/92
Age	Mean ± SE^$^ p* (vs. control)	60.3 ± 2.4 0.98	64.7 ± 1.3 0.12	60.3 ± 2.0	62.3 ± 1.4 0.84	62.7 ± 1.6	63.9 ± 0.6 < 0.01	52.8 ± 1.2
	Onset	36–73	46–77		36–76		36 – 83	

*: student’s *t*-test *p* < 0.05: statistically significant ^$^: age at sampling M: male F: female

**Fig. 2:**
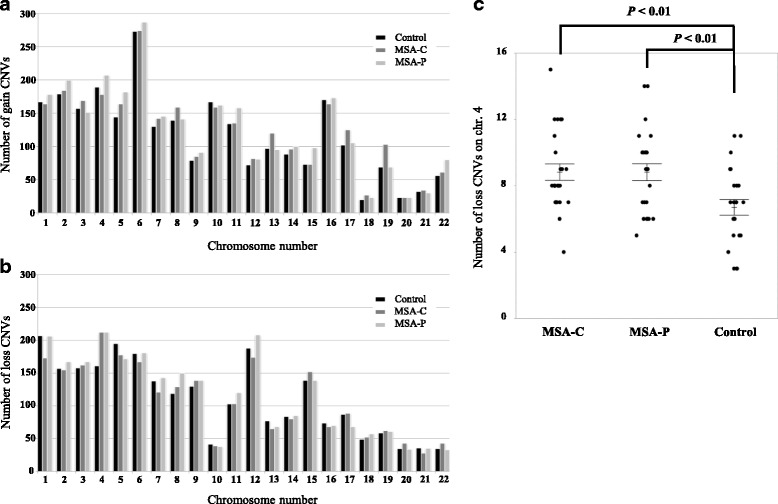
Number of CNVs in autosomal chromosomes in subjects with MSA-C or MSA-P and in controls. **a** Total number of gain CNVs in each chromosome (**b**) Total number of loss CNVs in each chromosome (**c**) Number of loss CNVs in chromosome 4 in subjects with MSA-C or MSA-P and in controls. Statistical significance was analyzed by the Wilcoxon rank-sum test. Error bars indicate ± standard error of the mean (SEM)

Moreover, we compared the distribution of 15,499 CNVs from chromosome 1 to chromosome 22 in MSA-C, MSA-P, and control subjects (Fig. 2a, b, and Additional file 1: Table S2a). Gain CNV numbers on chromosomes 2, 5, 11, 15, and 22 in MSA-P were significantly higher than those in the control group (chr.2: *p* = 0.04, chr.5: *p* < 0.01, chr.11: p = 0.02, chr.15: p = 0.02, chr.22: *p* < 0.01; Wilcoxon rank-sum test). Loss CNV numbers on chromosome 4 in MSA-P and MSA-C and chromosome 8 in MSA-P were significantly higher than those in the control group (chr.4: *p* < 0.01, chr.8: *p* = 0.03; Wilcoxon rank-sum test) (Fig. 2c). The number of gain CNVs was increased in MSA-P and that of loss CNVs on chromosome 4 was increased in all patients with MSA.

### CNVs related to MSA

Next, we analyzed CNVs related to MSA by comparing subjects with MSA and control subjects. Subjects in both MSA groups and the control group were matched for age and sex. MSA-C was more common than MSA-P in the Japanese population; therefore, we focused on MSA-C or all patients with MSA. We reanalyzed the microarray data of our 72 samples using the correlation aberration filter (see Materials and Methods for details). Furthermore, to identify CNVs related to MSA, reanalyzed data were filtered as follows in the 1st analysis: the length of the CNV was at least 1 kb; the number of subjects with MSA in which CNV was detected was more than twice that in controls. Based on these criteria, we endeavored to identify conservative CNVs associated with MSA. The aberration ratio on the X chromosome was compared between both males and females. We used CNV data for the X chromosome, but were unable to use CNV data for the Y chromosome. We identified a total of 379 CNVs in this step; of these, 216 were less than 10 kb and 163 were at least 10 kb in length. In the 2nd analysis, to evaluate CNVs of at least 10 kb in length in detail, we prepared an additional 80 Japanese genomic samples (40 unrelated patients with MSA and 40 unrelated adult healthy controls) and designed an Agilent 8x60K custom array with probe spacing of 205 bp in these CNV regions. The additional 80 samples did not overlap with the previous 72 samples. The details are described in the second set of Table 1. The custom arrays were assayed, and 163 CNVs of at least 10 kb in length were re-evaluated and identified. Custom array data were analyzed with the same filters as those used to analyze MSA-related CNVs. These CNVs were selected by increasing the number of samples analyzed, and the length of the regions was changed by short probe spacing during custom analysis. Consequently, they were classified as 30 CNVs less than 10 kb and 65 of at least 10 kb in length.

**Fig. 3:**
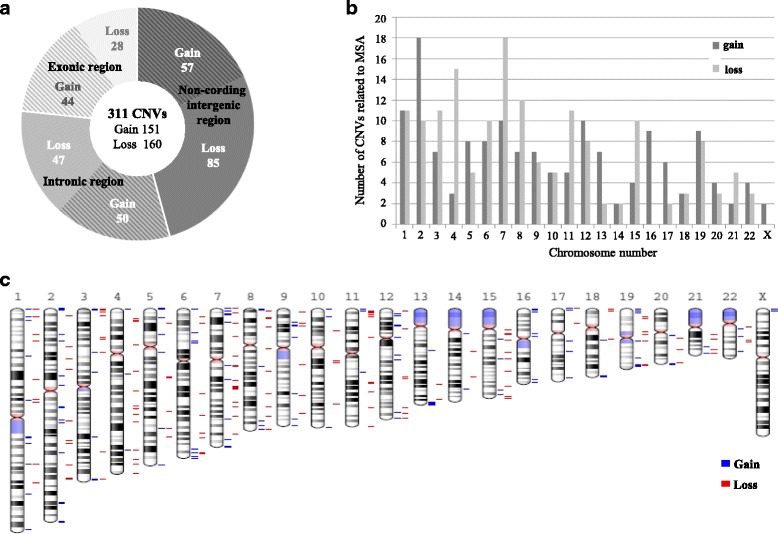
Description of 311 CNVs related to MSA. **a** Type and genomic region of 311 CNVs (**b**) Number of CNVs by type on each chromosome (**c**) Chromosomal location of 311 CNVs

Overall, we identified a total of 311 CNVs related to MSA, of which 246 (79.1%) were less than 10 kb and 65 (20.9%) were at least 10 kb in length. Only 13 CNVs were greater than 100 kb in length. Furthermore, of the 311 CNVs related to MSA, 142 (45.7%) were in non-coding intergenic regions and 169 (54.3%) were intragenic. Of the 169 CNVs located within gene regions, 72 (42.6%) were in exon-containing regions and 97 (57.4%) were in intronic regions (Fig. 3a). The number of gain and loss CNVs on chromosomes 1–22 was compared between the 311 CNVs related to MSA (Fig. 3b, Additional file 1: Table S3), and their chromosomal locations were determined (Fig. 3c). The number of loss CNVs was 5-fold larger than that of gain CNVs on chromosome 4, whereas only gain CNVs were detected on chromosomes 16 and X. CNVs related to MSA or CNVs of the controls were widely distributed on each chromosome, and were not particularly abundant in the telomere or centromere regions (Additional file 2: Figure S1).

**Fig. 4:**
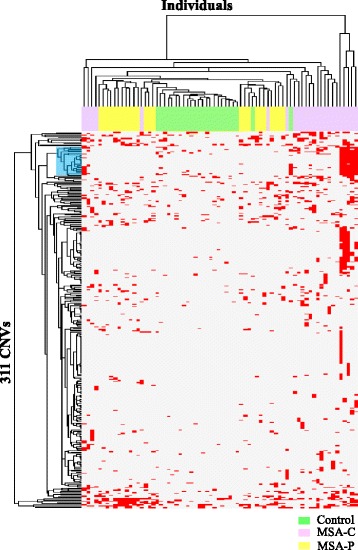
Cluster analysis of 311 CNVs related to MSA. Cluster heat map of relationships between 311 CNVs and 72 samples by cluster analysis; blue highlighting indicates a cluster of 29 CNVs

Moreover, we examined the relationships between the 311 CNVs and the samples by cluster analysis, and 29 CNVs were identified in 5 MSA-C samples (Fig. 4, Additional file 1: Table S4a). The age of onset of MSA-C in these patients ranged from 45 to 63 years; the ratio of males to females was 2: 3. We found no genetic similarity in the patients with the 29 CNVs.

### Gene set analysis of CNVs related to MSA

Of the 169 CNVs within genes, 72 were in exonic regions of 97 genes, and 97 CNVs were in intronic regions of 89 genes. To determine the relationships, pathways, and larger networks of the extracted CNVs, these 186 genes were evaluated using gene ontology (GO) analysis. A total of 11 GO sets were found by analysis of CNVs contained in exonic regions, with the functional categories significantly related to cell attachment and immune response. In contrast, 294 GO sets were observed via analysis of CNVs contained in intronic regions; these were involved in regulating gamma-aminobutyric acid secretion, D-aspartate transport, and synaptic transmission (Table 2). In addition, approximately 142 CNVs in non-coding intergenic regions were examined using the UCSC Genome Browser. Among these, 130 contained regions corresponding to retrotransposons, simple repeat sequences, CpG islands, large intergenic non-coding RNAs, and DNase I-hypersensitive sites. Only 12 CNVs were located in non-functional regions.

**Table 2:** GO process analysis of copy number variants (CNVs) related to MSA

GO set		Number of set genes	Number of CNV genes	*p*-value	*q*-value
(a) Results of GO process analysis with CNVs included in exonic regions					
GO:0007156	homophilic cell adhesion via plasma-membrane adhesion molecule	198	12	8.30e-11	1.71e-7
GO:0098742	cell-cell adhesion via plasma-membrane adhesion molecule	279	12	4.19e-9	4.31e-6
GO:0007155	cell adhesion	1706	24	3.23e-7	1.93e-4
GO:0022610	biological adhesion	1720	24	3.75e-7	1.93e-4
GO:0070423	nucleotide-binding oligomerization domain containing signaling pathway	41	5	1.04e-6	3.44e-4
GO:0035872	nucleotide-binding domain, leucine-rich repeat containing receptor signaling pathway	42	5	1.17e-6	3.44e-4
(b) Results of GO process analysis with CNVs in intronic regions					
GO:0014054	positive regulation of gamma-aminobutyric acid secretion	14	5	2.48e-9	5.48e-6
GO:0070777	D-aspartate transport	6	4	4.65e-9	5.48e-6
GO:0070779	D-aspartate import	6	4	4.65e-9	5.48e-6
GO:0014052	regulation of gamma-aminobutyric acid secretion	19	5	1.42e-8	7.39e-6
GO:0007268	chemical synaptic transmission	655	16	1.72e-8	7.39e-6
GO:0099537	trans-synaptic signaling	655	16	1.72e-8	7.39e-6

### Validation of CNVs related to MSA

**Fig. 5:**
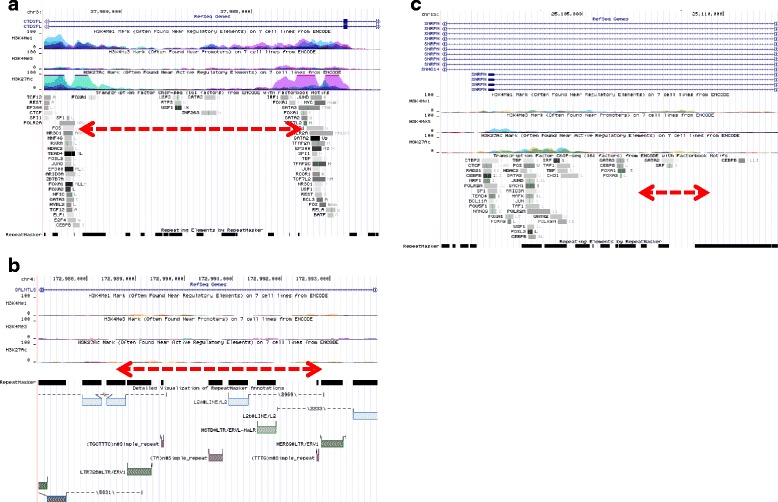
Genomic location of three CNVs. The genomic locations of three validated CNV, as displayed by the UCSC Genome Browser (NCBI Build 37/hg19), are shown. The indicated tracks contain information regarding transcriptional regulation (H3K4Me1, H3K4Me3, H3K27Ac, Txn Factor ChIP) from ENCODE. **a** *CTDSPL* (**b**) *GALNTL6* (**c**) *SNRPN*; the red dotted arrow indicates a genome loss region

To determine the frequency and position of extracted CNVs, we assayed the 457 Japanese genomic DNA samples by PCR. The details of the 457 individuals, including 152 whose samples were used for array analysis, are described as the validation set in Table 1. From among the 311 MSA-related CNVs mentioned above, we selected those with lengths of less than 10 kb and prepared a set of primers covering each candidate CNV. Among the selected regions, 42 CNVs were assayed and 12 loss CNVs were successfully verified. The successfully verified CNV data showed the same results by array-CGH and PCR. For these CNVs, an odds ratio of homozygous deletion, or both homozygous and heterozygous deletion, was calculated (Additional file 1: Table S5a). We identified high odds ratios for three CNVs of at least 2-fold higher in MSA than in controls. The three CNVs were located at 3p22.2, 4q34.1, and 15q11.2, and contained the CTD small phosphatase-like (*CTDSPL*), polypeptide *N*-acetylgalactosaminyltransferase-like 6 (*GALNTL6*), and small nuclear ribonucleoprotein polypeptide N (*SNRPN*) genes, respectively (Table 3, Additional file 2: Figure S2). These CNVs were present in the intronic regions of the genes and caused loss of the intronic region (Fig. 5). The positions of the lost regions completely matched between samples. However, no patients carried more than one of the three CNVs.

**Table 3:** Position and frequency of the three CNVs

Chr	Gene region	Position by sequencing			Control			MSA			Odds ratio for homo del (95% CI) (*p* value)
		Start	Deletion (bp) (Insert, nt)	Stop	No del	Hetero del	Homo del	No del	Hetero del	Homo del	
3	*CTDSPL*	37,978,418	8,510 (agg)	37,986,927	193	19	0	223	18	4	(0.025)
4	*GALNTL6*	172,988,640	4,292 (c)	172,992,931	133	75	4	156	79	10	2.21 (0.73–8.16) (0.166)
15	*SNRPN*	25,107,033	2,389 (atcatatcct)	25,109,421	194	18	0	219	23	3	(0.053)

No del: no deletion Hetero del: hetero deletion Homo del: homo deletion

## Discussion

In this study, we detected and verified CNVs using array-CGH and PCR, and we investigated the relationship between CNVs and MSA. We found that the number of gain CNVs was significantly increased on chromosomes 5 and 22 in patients with MSA-P, whereas the number of loss CNVs was significantly increased on chromosome 4 in all patients with MSA. Further, we identified 311 CNVs related to MSA and a characteristic cluster containing 29 CNVs. Finally, we detected 3 CNVs that may represent risk factors for MSA.

The genomic loci showing a significant increase in CNV numbers are 5p15.33, 5q35.3, 22q12.3, and 22q13.31 in MSA-P and 4q13.1, 4q28.3, 4q32.1, 4q34.1, and 4q35.2 in MSA. This increase in CNVs indicates an increase in genomic recombination events, although the overall recombination rate of these chromosomes was not particularly higher than that of other chromosomes. In addition, the loci of selective chromosomes in MSA-P did not completely match the loci of CNVs reported for Parkinson’s disease [30–32]. Thus, the increase in CNVs at the selected chromosome sites may be characteristic of MSA. Additionally, the increased number of CNVs in MSA was slightly greater in males than that in females (Additional file 1: Table S2b); however, previous studies have not observed any gender-related difference in the incidence of MSA [14–17]. Although various factors increase genomic instability, we cannot clearly explain the cause of the higher sensitivity of genomic instability in certain chromosomes in MSA or why the number of CNVs was slightly greater in males than that in females. In order to clarify these points, more detailed experiments on specific chromosomes and a large-scale familial survey will be necessary.

Large variants of several hundred kb in size affecting numerous genes have been reported as typical rare CNVs in several neurological and neurocognitive disorders [23, 26, 27, 33–35]. In contrast, small and mid-sized variants such as those less than 50 kb in size are regarded as common CNVs associated with disease [36, 37]. Most CNVs related to MSA were small variants of less than 10 kb in size, and only 13 CNVs were 100–500 kb in length (Additional file 1: Table S6). We speculate that the small CNVs are important in MSA because of their higher frequency.

The results of GO analyses indicated an association with MSA that differed from the results obtained in studies of other diseases [26, 38–40]. The GO results of CNVs in intronic regions were more closely related to the molecular mechanism of MSA than those of CNVs in exonic regions. Moreover, the GO results for 29 CNVs identified by cluster analysis indicated a relationship with oligodendrocytes, which play an important role in MSA [41, 42] (Additional file 1: Table S4b). Thus, the identified CNVs are likely related to the onset and pathology of MSA. Additional validation using a larger number of samples is necessary to confirm this hypothesis.

We identified three CNVs with allele frequencies that were significantly different between MSA and control samples. These CNVs were located in the gene bodies of *CTDSPL*, *GALNTL6*, and *SNRPN*. *CTDSPL* encodes a protein related to neuronal gene silencing in non-neuronal cells [43], *GALNTL6* encodes a protein related to cell maturation and differentiation in the brain and testis [44], and *SNRPN* encodes a protein that plays a role in pre-mRNA processing and tissue-specific alternative splicing events (associated with Prader-Willi syndrome) [45]. Although CNVs in exon-containing regions affect the expression of encoded proteins via a deletion or duplication mechanism, the three CNVs are located in intronic regions. However, these CNVs are contained in genomic regions containing elements such as transcription factor (TF)-binding sites, transposable elements (TEs), or a combination of these sites [46–48]. Deletion of a region by CNVs affects the structure of these elements. Recent reports showed that variations in TF-binding sites, TEs, and non-coding RNA lead to changes in gene expression [49–51] and cause disease [51–54]. Our study showed that approximately 77% of the identified CNVs were in non-coding regions; 95.0% of CNVs in non-coding regions contained active elements, such TF-binding sites, TEs, and non-coding RNA. Because the majority of the human genome comprises non-coding regions, these regions may be responsible for various functions that differ from those of protein-coding regions, which occupy only 1.5% of the human genome [55]. Structural changes in active elements of non-coding regions due to CNVs may affect disorder onset through modulation of gene expression or transcription. Thus, CNVs in non-coding regions are also likely involved in the pathogenesis and onset of MSA.

The detected CNVs in this study comprised similar common variants, with odds ratios between 0.86 and 1.47. We found that some patients had a combination of low-frequency CNVs, and that some combinations of CNVs increased the frequency and specificity of disease compared with the effect of each CNV alone. Prior to the clinical onset of MSA, a long preclinical process likely occurs in the molecular pathology of this disorder. The rate of progression of MSA is likely related to a combination of common CNVs each with small or moderate influence. Moreover, combinations of common CNVs with low frequency may cause non-heritable MSA.

Through CNV analysis, we showed that 1) the frequencies of CNVs were increased in selected chromosomes in MSA, 2) three CNVs potentially act as risk factors for MSA, and 3) CNVs in non-exonic, non-coding regions potentially affect the molecular process underlying MSA. As few studies have analyzed CNVs on a genome-wide scale with sufficient numbers of subjects, disease-associated polymorphisms in non-coding regions should be considered risk factors for MSA.

## Methods

### Participants and samples

Patients with MSA and control subjects were enrolled in the research study with approval by the Institutional Ethics Committee of Hokkaido University Faculty of Medicine and Graduate School of Medicine. All patients with MSA were neurologically evaluated by board-certified neurologists at the Department of Neurology, Hokkaido University Hospital, and at participating research institutes. The diagnosis of MSA was made based on the 2nd Consensus Criteria [3]. Written informed consent was obtained from all control subjects and all participants (or their families, when patients were unable to provide consent themselves). Among the patients, we selected 212 control subjects who were free from neurological disorders and 245 patients with MSA, and both groups donated blood samples for this study. Genomic DNA extracted from the peripheral blood samples was used in all experiments. We studied 48 patients with sporadic MSA (24 with MSA-C, 24 with MSA-P) and 24 healthy adult controls for genome-wide screening in the primary analysis. Additionally, in order to verify selected regions identified in the primary analysis, 40 patients with MSA-C and 40 healthy adult controls were chosen for secondary analysis using custom arrays. These were not matched to participants from the first analysis. For validation analysis, 245 patients with MSA (163 with MSA-C, 82 with MSA-P) and 212 healthy adult controls, including patients from the first and second analyses, were chosen. The age and gender of patients are listed in Table 1.

### Array-CGH assays

Genomic DNA was extracted from the buffy coat of peripheral blood using guanidine, and the samples were purified with 13% polyethylene glycol 6000 (Sigma-Aldrich, St. Louis, MO, USA) in 0.8 M NaCl. The concentration and purity of purified genomic DNA was measured using a Nano Drop 2000 (Thermo Fisher Scientific, Waltham, MA, USA). The concentration of double-strand DNA was measured using the Qubit® dsDNA BR assay with a Qubit® 3.0 fluorometer (Thermo Fisher Scientific). From these measurement results, the ratio of double-strand DNA was calculated, and genomic DNA with a value of at least 0.85 was used. Agilent Sure Print G3 Human CNV (2 × 400 K) arrays and Agilent Sure Print G3 Custom CGH Microarray (8 × 60 K) (Agilent Technologies, Santa Clara, CA, USA) were used for genome-wide array analysis. The Custom CGH Microarray was designed for regions identified to be at least 10 kb in length with the Human CNV (2 × 400 k) arrays using eArray (Agilent). All arrays were designed based on NCBI Build 37/UCSC hg19. Microarray analyses were performed following the manufacturer’s instructions, using 500 ng genomic DNA with a SureTag DNA Labeling Kit (Agilent). NA19000 (Coriell Institute, Camden, NJ, USA) was used as a reference for all microarray experiments. The microarrays were scanned with a Sure Scan Microarray Scanner (Agilent), and raw image data obtained by the scanner were processed using Feature Extraction Software (ver. 11.0.1.1, Agilent). The microarray data, quantified and processed, included quality-control data (QC Report) to ensure that the data was of high quality. According to the manufacturer’s recommendations, the derivative log ratio spread of the robust metric value of QC should be less than 0.30; the derivative log ratio spread of all genomic DNA passed QC with 0.11–0.17. The gender confirmed from the microarray data were all matched with the reported gender of patients whose DNA samples were analyzed.

### CNVs and GO analysis

The data processed by Feature Extraction Software were evaluated using the Aberration Detection Method 2 algorithm with a threshold of 6.0 with Agilent CytoGenomics software (ver. 2.7.8.0). To analyze individual arrays, the following standard aberration filters were used: minimum of three contiguous supra-threshold probes and minimum of 0.25 average log_2_ ratios. Moreover, to analyze CNVs related to MSA, the following correlation aberration filters were applied: minimum of two contiguous supra-threshold probes and minimum of 0.5 average log_2_ ratios. All arrays were designed based on NCBI Build 37/UCSC hg19. We used the UCSC Genome Browser for physical mapping and annotations of CNVs (http://genome.ucsc.edu/). If necessary, we also used data carried over to GRCh38 using the UCSC Genome Browser. For CNV analysis, we applied data for gene, interspersed repeats, or simple tandem repeats as categorized by the RepeatMasker, Simple Repeats, and large intergenic non-coding RNA tracks by the UCSC Genome Browser. In addition, we used integrated regulation data from the ENCODE track of the UCSC Genome Browser to obtain information relevant to transcriptional regulation. For GO process analysis, MetaCore™ (version 6.30, Thomson Reuters, New York, NY, USA) was used, and the significance levels were set at a *p* value of 0.01 and *q* (false discovery rate) of 0.01.

### PCR and sequencing

PCR primers were designed based on the results of array analysis (Additional file 1: Table S5b), and amplifications were performed using Tks Gflex™ DNA Polymerase (TaKaRa Bio, Inc., Shiga, Japan) and KOD FX Neo (Toyobo, Osaka, Japan). Amplification conditions are described in Additional file 1: Table S5c. PCR products were separated on a 0.8%, 1.0%, or 3% agarose gel and analyzed, and the PCR products were sequenced to detect deleted regions using the BigDye® Terminator v3.1 cycle Sequencing kit (Thermo Fisher Scientific).

### Statistical analysis

Statistical analysis was performed using JMP® Pro statistical software, version 12.0.1 (SAS Institute, Inc., Cary, NC, USA), and *p* values <0.05 were considered statistically significant. Cluster analysis was performed by complete-linkage hierarchical clustering with Hamming distance implemented in SciPy 0.18.1.

## **Additional files**

Additional file 1: Table S1. Number of CNVs in 72 individuals. **Table S2.** Number of CNVs on autosomal chromosomes in controls and subjects with MSA-C or MSA-P. **Table S3.** Chromosome numbers at which the 311 CNVs related to MSA were located. **Table S4.** Details of the 29 CNVs as obtained by cluster analysis. **Table S5.** Details of the 12 verified CNVs. **Table S6.** Large CNVs identified in this study. (DOCX 97 kb)

Additional file 2: Figure S1. Gel electrophoresis of PCR products for verification analysis. **Figure S2.** Chromosomal location of CNVs in control subjects. (PPTX 454 kb)

## Abbreviations

array-CGH: Array based comparative genomic hybridization
CNVs: Copy number variants
CTDSPL: CTD small phosphatase-like
GALNTL6: Polypeptide *N*-acetylgalactosaminyltransferase-like 6
GO: Gene ontology
MSA: Multiple system atrophy
MSA-C: Cerebellar type of MSA
MSA-P: Parkinsonian type of MSA
SNRPN: Small nuclear ribonucleoprotein polypeptide N
TEs: Transposable elements
TFs: Transcription factors
