# Supplementary material for: Genomic copy number variation analysis in multiple system atrophy
Source: Mol Brain. 2017 Nov 29;10:54. doi: 10.1186/s13041-017-0335-6 (PMC5708077; doi:10.1186/s13041-017-0335-6)
Supplement: Supplementary file 1 — Number of CNVs in 72 individuals. Table S2. Number of CNVs on autosomal chromosomes in controls and subjects with MSA-C or MSA-P. Table S3. Chromosome numbers at which the 311 CNVs related to MSA were located. Table S4. Details of the 29 CNVs as obtained by cluster analysis. Table S5. Details of the 12 verified CNVs. Table S6. Large CNVs identified in this study. (DOCX 97 kb) [file 13041_2017_335_MOESM1_ESM.docx]

Table S1. Number of CNVs in 72 individuals

**Number** **Control MSA-C MSA-P**

gain loss total gain loss total gain loss total

1 106 102 208 121 117 238 120 98 218

2 126 108 234 102 110 212 114 97 211

3 101 97 198 139 101 240 109 116 225

4 103 113 216 120 113 233 112 94 206

5 118 122 240 122 91 213 117 94 211

6 86 107 193 117 118 235 117 109 226

7 122 106 228 115 120 235 106 102 208

8 90 92 182 118 124 242 107 102 209

9 104 110 214 116 115 231 122 110 232

10 116 113 229 97 80 177 110 91 201

11 93 91 184 86 88 174 114 112 226

12 129 123 252 112 69 181 120 98 218

13 98 96 194 106 82 188 115 104 219

14 114 105 219 85 91 176 115 131 246

15 81 93 174 143 85 228 120 112 232

16 97 81 178 91 108 199 116 100 216

17 83 82 165 111 104 215 112 113 225

18 114 86 200 99 110 209 124 102 226

19 113 117 230 108 96 204 108 108 216

20 114 111 225 120 101 221 134 103 237

21 115 97 212 119 105 224 117 120 237

22 116 91 207 151 103 254 115 115 230

23 114 101 215 107 97 204 118 108 226

24 107 109 216 116 106 222 116 114 230 average 106.7 102.2 208.9 113.4 101.4 214.8 115.8 106.4 222.2

SD 13.3 11.8 22.2 16.2 14.0 23.0 6.1 9.4 11.0

*P** value

(vs. control)

0.14 0.93 0.34 **0.01** 0.22 **0.02**

*: Wilcoxon rank-sum test; *p< 0.05*

Table S2. Number of CNVs on autosomal chromosomes in controls and subjects with MSA-C or MSA-P

(a) Number of CNVs on chromosomes 1–22

| Chr | **Total** | | | | | **Gain** | | | | | **Loss** | | | | | | |
| --- | --- | --- | --- | --- | --- | --- | --- | --- | --- | --- | --- | --- | --- | --- | --- | --- | --- |
|  | Control | MSA-C | | MSA-P | | Control | MSA-C | | MSA-P | | Control | MSA-C | | | MSA-P | | |
|  |  |  | *p* value* (vs. cont) |  | *p* value* (vs. cont) |  |  | *p* value* (vs. cont) |  | *p* value* (vs. cont) |  |  | *p* value* (vs. cont) | |  | *p* value* (vs. cont) |  |
| 1 | 374 | 337 | 0.12 | 384 | 0.73 | 167 | 164 | 0.50 | 178 | 0.61 | 207 | 173 | 0.05 | | 206 | 0.93 |  |
| 2 | 336 | 339 | 0.68 | 366 | 0.21 | 179 | 184 | 0.76 | 199 | **0.04** | 157 | 155 | 0.88 | | 167 | 0.74 |  |
| 3 | 315 | 331 | 0.51 | 318 | 0.35 | 157 | 169 | 0.45 | 151 | 0.50 | 158 | 162 | 1.00 | | 167 | 0.68 |  |
| 4 | 350 | 390 | 0.19 | 419 | **< 0.01** | 189 | 178 | 0.62 | 207 | 0.20 | 161 | 212 | **< 0.01** | | 212 | **< 0.01** |  |
| 5 | 339 | 342 | 0.97 | 354 | 0.46 | 144 | 164 | 0.20 | 182 | **< 0.01** | 195 | 178 | 0.23 | | 172 | 0.15 |  |
| 6 | 453 | 441 | 0.74 | 468 | 0.41 | 273 | 274 | 0.81 | 287 | 0.29 | 180 | 167 | 0.74 | | 181 | 0.72 |  |
| 7 | 268 | 263 | 0.84 | 288 | 0.13 | 130 | 142 | 0.32 | 145 | 0.19 | 138 | 121 | 0.22 | | 143 | 0.44 |  |
| 8 | 258 | 288 | 0.10 | 291 | 0.10 | 139 | 159 | 0.12 | 141 | 0.92 | 119 | 129 | 0.37 | | 150 | **0.03** |  |
| 9 | 209 | 224 | 0.25 | 230 | 0.10 | 79 | 85 | 0.55 | 91 | 0.24 | 130 | 139 | 0.52 | | 139 | 0.50 |  |
| 10 | 209 | 198 | 0.41 | 200 | 0.42 | 167 | 159 | 0.55 | 162 | 0.7 | 42 | 39 | 0.82 | | 38 | 0.52 |  |
| 11 | 237 | 238 | 0.98 | 278 | **0.02** | 134 | 135 | 1.00 | 158 | **0.02** | 103 | 103 | 0.83 | | 120 | 0.20 |  |
| 12 | 260 | 256 | 0.83 | 289 | 0.07 | 72 | 82 | 0.56 | 81 | 0.14 | 188 | 174 | 0.36 | | 208 | 0.21 |  |
| 13 | 174 | 185 | 0.86 | 163 | 0.37 | 97 | 120 | 0.12 | 95 | 0.76 | 77 | 65 | 0.18 | | 68 | 0.33 |  |
| 14 | 172 | 176 | 0.91 | 185 | 0.21 | 88 | 96 | 0.42 | 100 | 0.11 | 84 | 80 | 0.66 | | 85 | 0.72 |  |
| 15 | 212 | 225 | 0.47 | 237 | 0.28 | 73 | 73 | 0.99 | 98 | **0.02** | 139 | 152 | 0.47 | | 139 | 0.92 |  |
| 16 | 244 | 232 | 0.29 | 243 | 0.96 | 170 | 164 | 0.60 | 173 | 0.97 | 74 | 68 | 0.40 | | 70 | 0.58 |  |
| 17 | 189 | 214 | 0.10 | 173 | 0.35 | 102 | 125 | 0.07 | 105 | 0.64 | 87 | 89 | 0.78 | | 68 | 0.13 |  |
| 18 | 69 | 79 | 0.19 | 80 | 0.42 | 20 | 27 | 0.25 | 23 | 1.00 | 49 | 52 | 0.55 | | 57 | 0.41 |  |
| 19 | 128 | 165 | 0.15 | 130 | 0.95 | 69 | 103 | 0.08 | 69 | 0.76 | 59 | 62 | 0.62 | | 61 | 0.41 |  |
| 20 | 58 | 66 | 0.23 | 57 | 0.93 | 23 | 23 | 1.00 | 23 | 1.00 | 35 | 43 | 0.19 | | 34 | 0.92 |  |
| 21 | 68 | 62 | 0.65 | 65 | 0.85 | 32 | 34 | 0.85 | 30 | 0.66 | 36 | 28 | 0.17 | | 35 | 0.85 |  |
| 22 | 91 | 104 | 0.10 | 113 | **0.02** | 56 | 61 | 0.31 | 80 | **< 0.01** | 35 | 43 | 0.20 | | 33 | 0.77 |  |
| total | 5,013 | 5,155 |  | 5,331 |  | 2,560 | 2,721 |  | 2,778 |  | 2,453 | 2,434 | |  | 2,553 |  |  |

*: Wilcoxon rank-sum test; *p* < 0.05

cont; control

(b) Number of gain and loss CNVs on chromosomes 4, 5, and 22.

| Chr | Sex | **Gain** | | | | | **Loss** | | | | |
| --- | --- | --- | --- | --- | --- | --- | --- | --- | --- | --- | --- |
|  |  | Control | MSA-C | | MSA-P | | Control | MSA-C | | MSA-P | |
|  |  |  |  | *p* value* (vs. cont) |  | *p* value* (vs. cont) |  |  | *p* value* (vs. cont) |  | *p* value* (vs. cont) |
| 4 | M | 97 | 89 | 0.54 | 94 | 0.93 | 72 | 110 | **< 0.01** | 108 | **< 0.01** |
|  | F | 92 | 89 | 1.00 | 113 | **0.03** | 89 | 102 | 0.31 | 104 | 0.29 |
| 5 | M | 66 | 82 | 0.22 | 93 | **0.01** | 97 | 94 | 0.77 | 91 | 0.79 |
|  | F | 78 | 82 | 0.61 | 89 | 0.13 | 98 | 84 | 0.15 | 81 | 0.13 |
| 22 | M | 28 | 31 | 0.63 | 44 | **0.04** | 17 | 26 | 0.05 | 18 | 1.00 |
|  | F | 28 | 30 | 0.34 | 36 | 0.09 | 18 | 17 | 1.00 | 15 | 0.56 |

*: Wilcoxon rank-sum test; *p* < 0.05

Table S3. Chromosome numbers at which the 311 CNVs related to MSA were located

| Chr | Number of CNVs | | |
| --- | --- | --- | --- |
|  | gain | loss | total |
| 1 | 11 | 11 | 22 |
| 2 | 18 | 10 | 28 |
| 3 | 7 | 11 | 18 |
| 4 | 3 | 15 | 18 |
| 5 | 8 | 5 | 13 |
| 6 | 8 | 10 | 18 |
| 7 | 10 | 18 | 28 |
| 8 | 7 | 12 | 19 |
| 9 | 7 | 6 | 13 |
| 10 | 5 | 5 | 10 |
| 11 | 5 | 11 | 16 |
| 12 | 10 | 8 | 18 |
| 13 | 7 | 2 | 9 |
| 14 | 2 | 2 | 4 |
| 15 | 4 | 10 | 14 |
| 16 | 9 | 0 | 9 |
| 17 | 6 | 2 | 8 |
| 18 | 3 | 3 | 6 |
| 19 | 9 | 8 | 17 |
| 20 | 4 | 3 | 7 |
| 21 | 2 | 5 | 7 |
| 22 | 4 | 3 | 7 |
| X | 2 | 0 | 2 |

　Table S4. Details of the 29 CNVs as obtained by cluster analysis

(a) Set of 29 CNVs from cluster heat map

| **Chr** | **Locus** | **Start** | **Stop** | **Size (bp)** | **Type** | **Region** | **Gene** |
| --- | --- | --- | --- | --- | --- | --- | --- |
| 13 | q34 | 114,881,621 | 114,883,633 | 2,012 | Gain | intron | *RASA3* |
| 19 | q12 | 29,619,177 | 29,621,107 | 1,930 | Gain | intergenic |  |
| 7 | q11.21 | 64,894,584 | 64,896,266 | 1,682 | Gain | intergenic |  |
| 13 | q34 | 111,133,066 | 111,135,249 | 2,183 | Gain | exon | *COL4A2* |
| X | p22.33 | 688,815 | 690,381 | 1,566 | Gain | intergenic |  |
| 6 | p25.3 | 519,789 | 522,032 | 2,243 | Gain | intron | *EXOC2* |
| 19 | q13.42 | 56,071,470 | 56,075,620 | 4,150 | Gain | intergenic |  |
| 2 | q37.3 | 241,979,159 | 241,980,279 | 1,120 | Gain | exon | *SNED1* |
| 16 | p13.3 | 2,388,441 | 2,389,888 | 1,447 | Gain | intron | *ABCA3* |
| 22 | q13.31 | 45,140,279 | 45,141,517 | 1,238 | Gain | intron | *PRR-ARHGAP8* |
| 6 | p21.2 | 37,663,196 | 37,664,292 | 1,096 | Gain | intron | *MDGA1* |
| 1 | p36.22 | 10,754,797 | 10,756,794 | 1,997 | Gain | intron | *CASZ1* |
| 12 | q24.31 | 124,807,424 | 124,809,278 | 1,854 | Gain | exon | *NCOR2* |
| 12 | q13.3 | 57,943,168 | 57,945,035 | 1,867 | Gain | exon | *KIF5A* |
| 16 | q24.1 | 85,186,979 | 85,189,242 | 2,263 | Gain | intergenic |  |
| 19 | p13.3 | 500,078 | 504,651 | 4,573 | Gain | exon | *MADCAM1* |
| 15 | q26.1 | 91,399,788 | 91,401,124 | 1,336 | Gain | intergenic |  |
| 2 | p25.3 | 305,342 | 306,449 | 1,107 | Gain | intergenic |  |
| 12 | q13.13 | 54,439,844 | 54,441,104 | 1,260 | Gain | intron | *HOXC4* |
| 16 | p13.3 | 2,566,577 | 2,568,274 | 1,697 | Gain | intron | *ATP6V0C* |
| 1 | q32.1 | 204,437,244 | 204,438,564 | 1,320 | Gain | exon | *PIK3C2B* |
| 5 | p15.33 | 51,015 | 52,684 | 1,669 | Gain | intergenic |  |
| 19 | q13.42 | 53,605,867 | 53,607,766 | 1,899 | Gain | exon | *ZNF160* |
| 17 | p13.3 | 912,162 | 913,389 | 1,227 | Gain | exon | *ABR* |
| 20 | q13.33 | 62,900,482 | 62,902,894 | 2,412 | Gain | intron | *PCMTD2* |
| 11 | p15.5 | 979,305 | 981,269 | 1,964 | Gain | intron | *AP2A2* |
| 6 | p22.3 | 19,953,396 | 19,956,241 | 2,845 | Loss | intergenic |  |
| 3 | q29 | 195,471,651 | 195,477,001 | 5,350 | Loss | exon | *MUC4* |
| 3 | q29 | 195,778,708 | 195,780,339 | 1,631 | Loss | intron | *TFRC* |

(b) Results of GO process analysis for the 29 CNVs

| GO set | | Number of set genes | Number of CNV genes | *p* value | *q*-value |
| --- | --- | --- | --- | --- | --- |
|  |  |  |  |  |  |
| GO:0019886 | antigen processing and presentation of exogenous peptide antigen via MHC class II | 171 | 4 | 2.45E-05 | 9.34E-03 |
| GO:0002495 | antigen processing and presentation of peptide antigen via MHC class II | 173 | 4 | 2.57E-05 | 9.34E-03 |
| GO:0002504 | antigen processing and presentation of peptide or polysaccharide antigen via MHC class II | 178 | 4 | 2.87E-05 | 9.34E-03 |
| GO:0007169 | transmembrane receptor protein tyrosine signaling pathway | 664 | 6 | 4.02E-05 | 9.34E-03 |
| GO:0038063 | collagen-activated tyrosine kinase receptor signaling pathway | 2 | 10 | 4.39E-05 | 9.34E-03 |

Table S5. Details of the 12 verified CNVs

| **Chr** | **Locus** | **Start** | **Stop** | **Size (bp)** | **Type** | **Region** | **Gene** | **Odds ratio (95% CI)** | ***p* value** |
| --- | --- | --- | --- | --- | --- | --- | --- | --- | --- |
| 1 | p13.3 | 108,733,653 | 108,739,610 | 5,958 | loss | intron / exon | *SLC25A24* | 0.89 (0.55–1.44) | 0.636 |
| 2 | q23.3 | 154,896,483 | 154,898,756 | 2,274 | loss | intron | *GALNT13* | 1.24 (0.27–6.38) | 0.776 |
| 2 | p24.1 | 19,767,153 | 19,771,534 | 4,382 | loss | intergenic | non-coding | 1.04 (0.31–3.65) | 0.950 |
| 3 | p22.2 | 37,978,371 | 37,986,984 | 8,614 | loss | intron | *CTDSPL* |  | 0.025 |
| 4 | q34.1 | 172,988,641 | 172,992,931 | 4,291 | loss | intron | *GALNTL6* | 2.21 (0.73–8.16) | 0.166 |
| 6 | q22.32 | 126,183,401 | 126,186,543 | 3,143 | loss | intron | *NCOA7* | 0.86 (0.43–1.71) | 0.670 |
| 6 | q24.1 | 139,602,408 | 139,607,245 | 4,838 | loss | intron | *TXLNB* | 1.45 (0.35–7.14) | 0.609 |
| 8 | q13.3 | 72,214,719 | 72,217,537 | 2,819 | loss | intron | *EYA1* | 1.31 (0.37–5.16) | 0.681 |
| 10 | p12.31 | 19,996,410 | 19,998,626 | 2,217 | loss | intron | *MALRD1* | 1.08 (0.55–2.12) | 0.831 |
| 15 | q11.2 | 25,107,308 | 25,118,365 | 11,058 | loss | intron | *SNRPN* |  | 0.053 |
| 21 | q21.1 | 19,323,772 | 19,329,870 | 6,099 | loss | intron | *CHODL* | 1.29 (0.71–2.38) | 0.406 |
| 22 | q12.3 | 33,757,415 | 33,760,045 | 2,631 | loss | intron | *LARGE1* | 1.47* (0.64–3.57) | 0.366* |

(a) Array regions of 12 CNVs and results of verification analysis

)

*: Odds ratio when including hetero deletion

(b) Primer set for verification of the 12 CNVs

| **Locus** | **Forward primer** | **Reverse primer** | **Reference product length (bp)** |
| --- | --- | --- | --- |
| 1p13.3 | 5'-CAGCATTCTGTGGCGCCATTTACG-3' | 5'-AGAGGCATGTCAGGGGTTTATGTAATG-3' | 9027 |
| 2q23.3 | 5'-AGTTTGCCAGCGATTAGTGTC-3' | 5'-GTGATACCTGTCTTCTCCTTACTC-3' | 8930 |
| 2p24.1 | 5'-TACTGTGGGTCAACTAAGCAGG-3' | 5'-CTGCTAAATGCTGCTCAACTGG-3' | 4954 |
| 3p22.2 | 5'-GTAGCATTAACAGCATTGATGTCC-3' | 5'-CTGTAGGTCCCGATAGTTCACAA-3' | 8695 |
| 4q34.1 | 5'-ACCTTTGACCTCCGATACTGAAC-3' | 5'-GAACCGCACTCTTGGCATTG-3' | 8935 |
| 6q22.32 | 5'-GCCCAACACTTTTCCATACACAG-3' | 5'-CCCAGTGCACAAAATGGAGATAC-3' | 5335 |
| 6q24.1 | 5'-TGGAACCACCTGTGTAGCAG-3' | 5'-TAACCAGACTATCTGTAAACCCAA-3' | 7558 |
| 8q13.3 | 5'-GCTGGATACGGTGAGCTGTT-3' | 5'-CCCACGATCAGTCACACTCC-3' | 7140 |
| 10p12.31 | 5'-TACGAACAAGGCAAGTGCTCTAA-3' | 5'-CCCTAAGGATGCATTTAAGGAGAA-3' | 3780 |
| 15q11.2 | 5'-GGAGAGCAACTTGATACAAACTGG-3' | 5'-TCTTAACACCCACATACATGTGTCT-3' | 8506 |
| 21q21.1 | 5'-AGCATGCTGCTATGATTTTGAATA-3' | 5'-TGCAACCGAAGAATAGACTGACA-3' | 5810 |
| 22q12.3 | 5'-AGGGGCTCTGTTCTGGAGTTA-3' | 5'-ACGCGGAACGGGATTGAAAA-3' | 5369 |

(c) Amplification conditions of 12 CNV verifications

| **Locus** | **Gene** | **Step** | | | **Cycle** | **Polymerase** |
| --- | --- | --- | --- | --- | --- | --- |
| 1p13.3 | *SLC25A24* | 98ºC 10 s | 68ºC 6.5 min | | 30 | Tks Gflex^TM^ |
| 2q23.3 | *GALNT13* | 98ºC 10 s | 68ºC 6.5 min | | 30 | Tks Gflex^TM^ |
| 2p24.1 | non-cording | 98ºC 10 s | 68ºC 3.0 min | | 30 | KOD FX Neo |
| 3p22.2 | *CTDSPL* | 98ºC 10 s | 60ºC 30 s | 68ºC 10 min | 35 | Tks Gflex^TM^ |
| 4q34.1 | *GALNTL6* | 98ºC 10 s | 68ºC 6.5 min | | 30 | Tks Gflex^TM^ |
| 6q22.32 | *NCOA7* | 98ºC 10 s | 60ºC 15 s | 72ºC 6 min | 35 | Tks Gflex^TM^ |
| 6q24.1 | *TXLNB* | 98ºC 10 s | 63ºC 10 s | 68ºC 8 min | 30 | KOD FX Neo |
| 8q13.3 | *EYA1* | 98ºC 10 s | 68ºC 4 min | | 30 | KOD FX Neo |
| 10p12.31 | *MALRD1* | 98ºC 10 s | 61ºC 10 s | 68ºC 2 min | 30 | KOD FX Neo |
| 15q11.2 | *SNRPN* | 98ºC 10 s | 61ºC 10 s | 68ºC 9 min | 30 | KOD FX Neo |
| 21q21.1 | *CHODL* | 98ºC 10 s | 61ºC 15 s | 68ºC 5 min | 30 | Tks Gflex^TM^ |
| 22q12.3 | *LARGE1* | 98ºC 10 s | 68ºC 6 min | | 30 | Tks Gflex^TM^ |

Table S6. Large CNVs identified in this study

| **Chr** | **Locus** | **Start** | **Stop** | **Size (bp)** | **Type** | **Region** |
| --- | --- | --- | --- | --- | --- | --- |
| 1 | q21.1 | 145,626,237 | 145,746,971 | 120,735 | gain | *NBPF10*, *NBPF19*, *NBPF20*, *RNF115*, *CD160*, *PDZK1* |
| 1 | q31.1 | 189,326,032 | 189,544,183 | 218,152 | loss | Non-coding |
| 2 | q13 | 110,852,960 | 110,983,703 | 130,744 | gain | *MALL, NPHP1*, lincRNA |
| 3 | p26.3 | 1,495,259 | 1,596,582 | 101,324 | gain | non-coding |
| 6 | q25.3 | 158,619,259 | 158,767,706 | 148,448 | gain | *GTF2H5*, *TULP4* |
| 7 | p22.2-p22.1 | 4,253,986 | 4,691,088 | 437,103 | gain | *SDK1* |
| 7 | p21.2 | 14,657,655 | 14,999,213 | 341,559 | gain | *DGKB* |
| 7 | q21.11 | 81,918,507 | 82,124,733 | 206,227 | gain | *CACNA2D1* |
| 7 | q31.33 | 125,227,215 | 125,406,147 | 178,933 | loss | non-coding |
| 12 | p13.31 | 7,996,763 | 8,125,276 | 128,514 | gain | *SLC2A14*, *SLC2A3* |
| 20 | q11.21 | 29,833,386 | 29,998,926 | 165,541 | gain | *DEFB115*, *DEFB116*, *DEFB118*, *DEFB119*, *DEFB121* |
| 21 | q21.2 | 25,388,477 | 25,767,317 | 378,841 | loss | non-coding, lincRNA |
| X | p22.33 | 2,278,247 | 2,418,245 | 139,999 | gain | *DHRSX*, *ZBED1* |
