# Supplementary material for: Genomic copy number variation analysis in multiple system atrophy
Source: Mol Brain. 2017 Nov 29;10:54. doi: 10.1186/s13041-017-0335-6 (PMC5708077; doi:10.1186/s13041-017-0335-6)
Supplement: Supplementary file 2 — Gel electrophoresis of PCR products for verification analysis. Figure S2. Chromosomal location of CNVs in control subjects. (PPTX 454 kb) [file 13041_2017_335_MOESM2_ESM.pptx]

## Slide 1
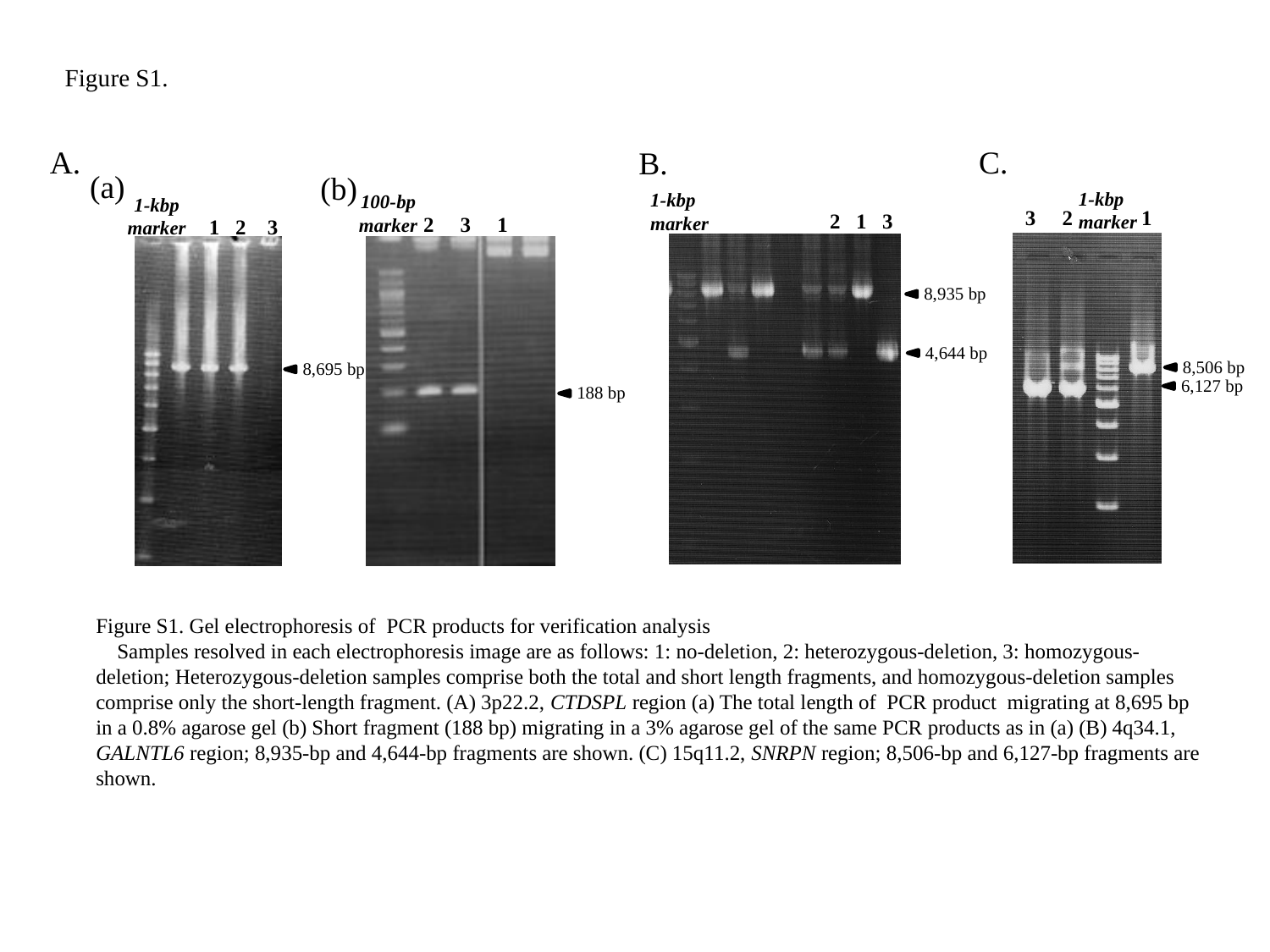

Figure S1.
C.
A.
B.
(a)
(b)
1-kbp marker
3 2 1
1-kbp marker
2 1 3
100-bp marker
2 3 1
1-kbp marker
1 2 3
8,935 bp
4,644 bp
8,506 bp
8,695 bp
6,127 bp
188 bp
Figure S1. Gel electrophoresis of PCR products for verification analysis
 Samples resolved in each electrophoresis image are as follows: 1: no-deletion, 2: heterozygous-deletion, 3: homozygous-deletion; Heterozygous-deletion samples comprise both the total and short length fragments, and homozygous-deletion samples comprise only the short-length fragment. (A) 3p22.2, CTDSPL region (a) The total length of PCR product migrating at 8,695 bp in a 0.8% agarose gel (b) Short fragment (188 bp) migrating in a 3% agarose gel of the same PCR products as in (a) (B) 4q34.1, GALNTL6 region; 8,935-bp and 4,644-bp fragments are shown. (C) 15q11.2, SNRPN region; 8,506-bp and 6,127-bp fragments are shown.

## Slide 2
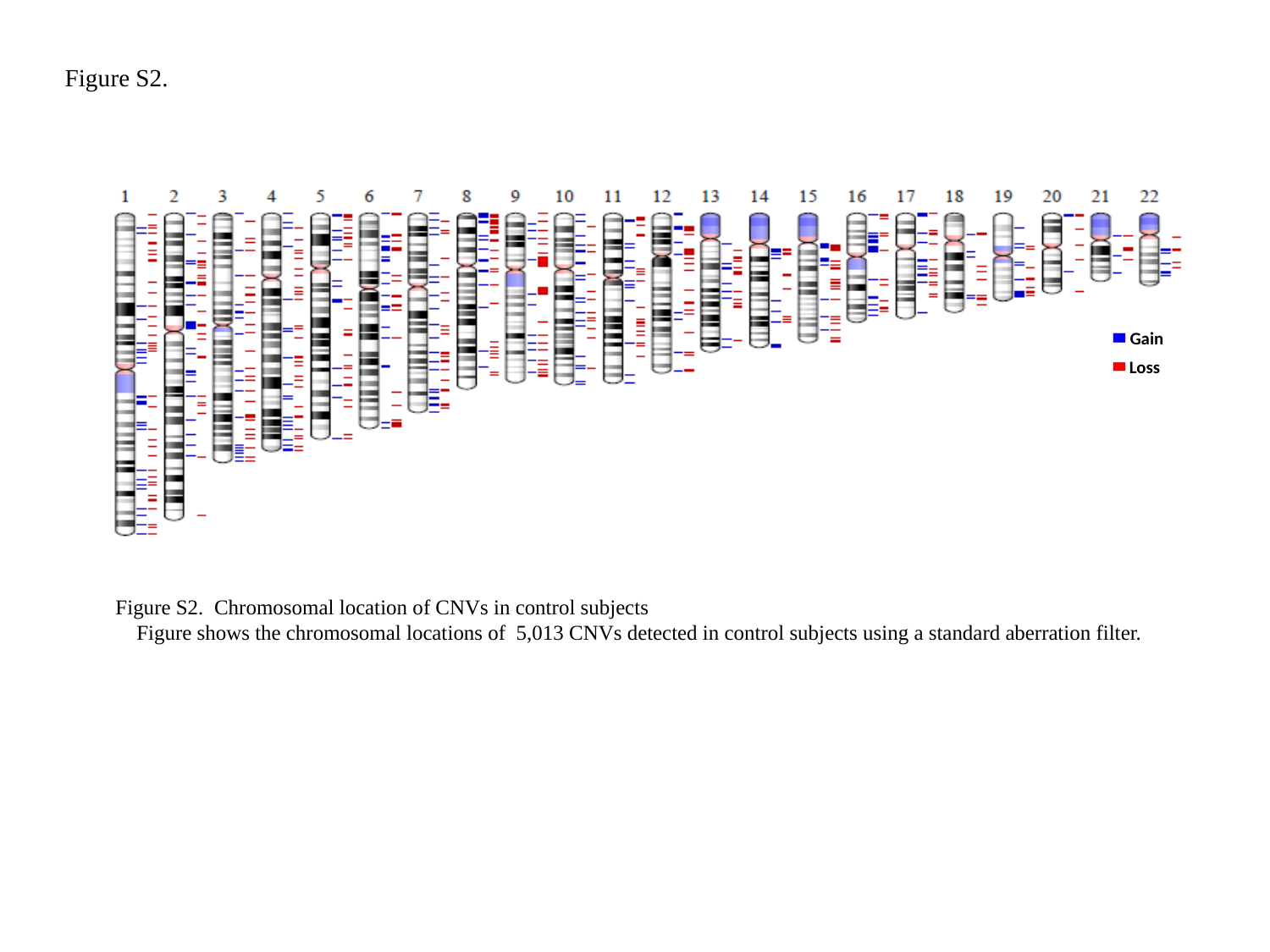

Figure S2.
Gain
Loss
Figure S2. Chromosomal location of CNVs in control subjects
 Figure shows the chromosomal locations of 5,013 CNVs detected in control subjects using a standard aberration filter.
